# Comparative outcomes of SARS-CoV-2 primary and reinfection in older adult patients

**DOI:** 10.3389/fpubh.2024.1337646

**Published:** 2024-02-16

**Authors:** Shu-Farn Tey, Ya-Wen Tsai, Jheng-Yan Wu, Ting-Hui Liu, Min-Hsiang Chuang, Wan-Hsuan Hsu, Po-Yu Huang, Chih-Cheng Lai, Chi-Kuei Hsu

**Affiliations:** ^1^Department of Medical Laboratory Science and Biotechnology, Chung Hwa University of Medical Technology, Tainan, Taiwan; ^**2**^Division of Pulmonary Medicine, Chi-Mei Medical Center, Tainan, Taiwan; ^3^Center for Integrative Medicine, Chi Mei Medical Center, Tainan City, Taiwan; ^4^Department of Medical Laboratory Sciences and Biotechnology, Fooyin University, Kaohsiung, Taiwan; ^5^Department of Nutrition, Chi Mei Medical Center, Tainan, Taiwan; ^6^Graduate Institute of Medicine, College of Medicine, Kaohsiung Medical University, Kaohsiung, Taiwan; ^7^Department of Psychiatry, Chi Mei Medical Center, Tainan, Taiwan; ^8^Department of Internal Medicine, Chi Mei Medical Center, Tainan, Taiwan; ^9^Division of Hospital Medicine, Department of Internal Medicine, Chi Mei Medical Center, Tainan, Taiwan; ^10^School of Medicine, College of Medicine, National Sun Yat-sen University, Kaohsiung, Taiwan; ^11^Department of Internal Medicine, E-Da Hospital, I-Shou University, Kaohsiung, Taiwan; ^12^School of Medicine for International Students, College of Medicine, I-Shou University, Kaohsiung, Taiwan

**Keywords:** COVID-19, outcome, primary infection, reinfection, SARS-CoV-2

## Abstract

**Background:**

The outcomes of older adult people acquiring SARS-CoV-2 reinfection was unclear. This study aimed to compare the outcomes of older adult patients with COVID-19 reinfection and those with primary infection.

**Methods:**

This retrospective cohort study used electronic medical records from the TriNetX Research Network. Older adult patients (aged ≥65 years) with COVID-19 between January 1, 2022, and December 31, 2022, were included in the study. The patients were subsequently categorized into reinfection or primary infection groups, according to whether they manifested two distinct COVID-19 episodes with an intervening period of more than 90 days. Propensity score matching was performed for covariate adjustment between the reinfection and primary infection groups. The primary outcome was a composite outcome, including emergency department visits, hospitalization, intensive care unit admission, mechanical ventilation use, and mortality, following primary infection and reinfection.

**Results:**

After matching, 31,899 patients were identified in both the reinfection and primary infection groups. The risk of primary composite outcomes was 7.15% (*n* = 2,281) in the reinfection group and 7.53% (*n* = 2,403) in the primary infection group. No significant difference in the primary outcome was observed between groups (HR, 0.96; 95% CI, 0.91 to 1.02, *p* = 0.17). In addition, there was no significant differences between the reinfection and primary infection groups in terms of emergency department visit (HR, 1.03; 95% CI, 0.95 to 1.11, *p* = 0.49), all-cause hospitalization (HR, 0.94; 95% CI, 0.86 to 1.02, *p* = 0.14), intensive care unit admission (HR, 0.92; 95% CI, 0.67 to 1.28, *p* = 0.62), mechanical ventilation use (HR,1.35 95% CI, 0.69 to 2.64 *p* = 0.38), and all-cause mortality (HR, 0.94; 95% CI, 0.74 to 1.20, *p* = 0.62).

**Conclusion:**

There were no significant differences in clinical outcomes between older adult patients with COVID-19 reinfection and those with primary infection.

## 1 Introduction

Since the first outbreak of SARS-CoV-2 infection at the end of 2019, more than 770 million confirmed cases of COVID-19 have been reported to the World Health Organization (1). To combat this global health threat, the implementation of non-pharmacological interventions and widespread vaccination programs have helped prevent the transmission of SARS-CoV-2 (2, 3). Additionally, the development of novel antiviral and other anti-COVID-19 treatments has been effective in improving patient outcomes (4–6). However, SARS-CoV-2 has been constantly evolving during the pandemic, giving rise to new variants with immune escape characteristics. These variants not only possess the ability to evade neutralizing antibodies but can also overcome immune protection following natural infection. Consequently, the emergence of SARS-CoV-2 reinfection has become a serious threat and raised new global health concerns. Older adult people, especially those with comorbidities, have a higher risk of acquiring SARS-CoV-2 reinfection (7–9). Moreover, age is a significant risk factor for COVID-19 progression (3, 10, 11). However, the outcomes in older adult patients with SARS-CoV-2 reinfection are poorly understood.

A meta-analysis including 52 studies between 2019 and 2022 reported that the overall prevalence of SARS-CoV-2 reinfection was 4.2% (95% confidence interval [CI]: 3.7–4.8%); however, high heterogeneity was observed between studies (I^2^ = 99.9%) (12). Another meta-analysis involving 11 studies and 11 case reports showed that the pooled SARS-CoV-2 reinfection incidence rate was 0.70 (standard deviation [SD], 0.33) per 10,000 person-days (13). Furthermore, a cohort study utilizing the United States Department of Veterans Affairs’ national healthcare database found that SARS-CoV-2 reinfection was associated with an increased risk of death, hospitalization, and the likelihood of experiencing at least one sequela (14). In contrast, another nationwide study conducted in the United Kingdom showed that reinfection with SARS-CoV-2 presented with milder symptoms and carried a lower risk of COVID-19-related hospitalization and intensive care unit (ICU) admission compared to primary infection (15). To resolve these conflicting findings, this study using a global database – TriNetX research network, was conducted to compare the short-term outcome of SARS-CoV-2 reinfection compared with primary infection among the older adult patients, who are at high risk of COVID-19 progression.

## 2 Methods

### 2.1 Data source

We extracted data from the TriNetX Research Network, a global collaborative clinical research platform that contains real-time electronic medical data collected from 110 million patients from 78 healthcare organizations (HCOs) across four countries. Diagnoses based on the International Classification of Diseases, Tenth Revision, Clinical Modification (ICD-10-CM) were retrieved from the TriNetX platform. This study was approved by the Institutional Review Board of the Chi Mei Medical Center (No. 11202–002).

### 2.2 Patient selection

Older adult patients (aged ≥65 years) with more than two visits to the HCOs and a COVID-19 diagnosis between January 1, 2022, and December 31, 2022, were included in the study. The ICD-10-CM diagnostic codes (U07.1, J12.81, and J12.82), positive SARS-CoV-2 RNA test (LOINC 94309–2, 94,500–6, 95,406–5, 94,502–2, 94,565–9, 95,608–6, 94,759–8, 94,845–5), and COVID-19 antigen test by immunoassay (LOINC 94558–4, 96,119–3) were used to extract the COVID-19 cohort. Patients were excluded from the analysis if they were admitted to a hospital from 3 days before to 2 days after the COVID diagnosis, or if they died on the same day as their COVID diagnosis. The patients were subsequently categorized into reinfection or primary infection groups according to whether they manifested two distinct COVID-19 episodes with an intervening period of more than 90 days.

### 2.3 Covariates

A 1:1 propensity score matching (PSM) through the greedy nearest-neighbor algorithm was performed using the TriNetX built-in platform for covariate adjustment. Demographic characteristics (age, sex, and race) and comorbidities (body mass index, primary hypertension [ICD-10-CM E10], neoplasms [C00-D49], hyperlipidemia [E78.5], diabetes mellitus [E08-E13], atherosclerotic heart disease [I25.1], heart failure [I50], transplantation, and immunosuppressive therapy) were used to minimize confounding factors and selection bias, as previous studies (16–18). A standard difference of <0.1 was considered an acceptable balanced match.

### 2.4 Outcomes

The primary composite outcomes were all-cause emergency department (ED) visits, hospitalization, utilization of intensive care (ICU), utilization of mechanical ventilation (MV), or death during the 30-day follow-up period. The secondary outcomes were the individual components of the primary outcomes: all-cause hospitalization, ED visits, intensive care utilization, mechanical ventilation utilization, and death.

### 2.5 Statistical analysis

All statistical analyses were performed using TriNetX built-in function. Baseline characteristics were delineated as either mean ± standard deviation or specific frequency and proportion. Hazard ratios (HRs) with 95% confidence intervals (CIs) were derived using Cox proportional hazard regression. Kaplan–Meier curves facilitated the determination of cumulative probability, with a value of p of less than 0.05 deemed statistically significant. Moreover, subgroup analyses were performed based on age, sex, comorbidities, vaccination status, and use of antiviral medication.

## 3 Results

### 3.1 Demographic characteristics of the enrolled patients

**Figure 1:**
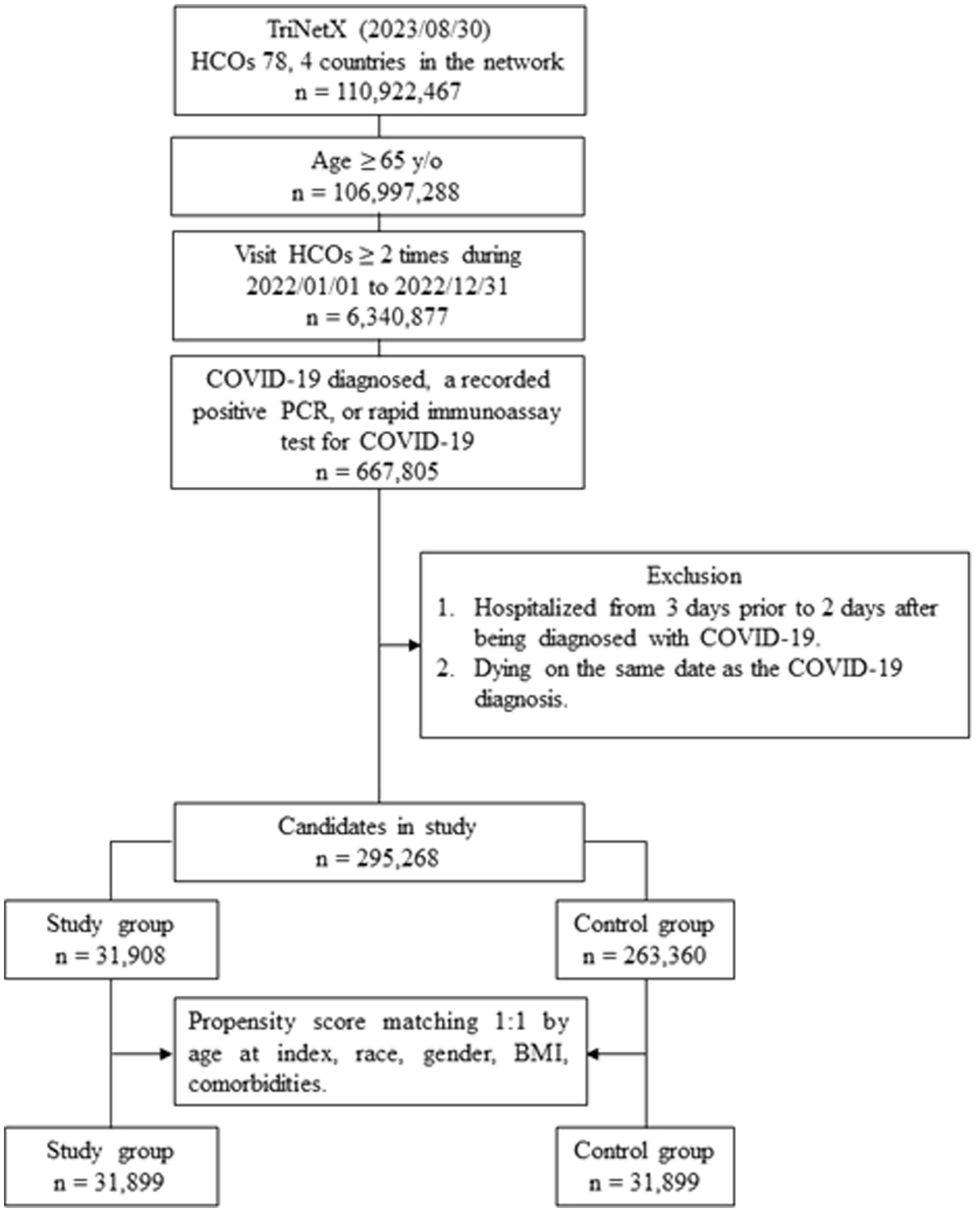
The algorithm of COVID-19 reinfection and primary infection cohort establishment.

The initial screening was conducted on August 30, 2023, identifying 295,268 older adult patients with COVID-19 from 78 healthcare organizations across the four countries (Figure 1). Among them, 31,908 had reinfections and 263,360 patients had primary infection. After PSM, 31,899 patients were identified in both the reinfection and primary infection groups.

Before matching, there were no significant differences in age, sex, or race between the reinfection and primary infection groups. However, patients with reinfection had a higher BMI than the primary infection group (29.3 ± 6.2 vs. 28.5 ± 5.9, *p* < 0.0001) and a higher prevalence of comorbidities, including hypertension, hyperlipidemia, neoplasms, diabetes mellitus, ischemic heart diseases, chronic lower respiratory diseases, overweight/obesity, cerebrovascular diseases, asthma, chronic liver disease, and pulmonary heart disease, as well as a higher percentage receiving immunosuppressants compared to those with primary infection. After PSM, there were no significant differences in the demographic features, including comorbidities, between the groups (Table 1).

**Table 1:** Baseline characteristics of study population (before and after PSM matching).

Variable	Before matching			After matching		
	Reinfection (*n* = 31,908)	Primary infection (*n* = 263,360)	Std diff	Reinfection (*n* = 31,899)	Primary infection (*n* = 31,899)	Std diff
Age at index, years						
Mean ± SD	73.0 ± 7.2	73.5 ± 7.3	0.07	73.0 ± 7.2	72.9 ± 7.0	0.02
Gender, n (%)						
Female	18,194 (57)	149,397 (56.7)	0.01	18,189 (57)	18,149 (56.9)	<0.001
Male	13,585 (42.6)	112,931 (42.9)	0.01	13,581 (42.6)	13,581 (42.6)	<0.001
Race, n (%)						
White	24,110 (75.6)	199,479 (75.7)	0.00	24,107 (75.6)	24,534 (76.9)	0.03
Black or African American	3,040 (9.5)	22,914 (8.7)	0.03	3,037 (9.5)	2,843 (8.9)	0.02
Asian	739 (2.3)	9,555 (3.6)	0.08	739 (2.3)	683 (2.1)	0.01
Body Mass Index, kg/m^2^						
Mean ± SD	29.3 ± 6.2	28.5 ± 5.9	0.13	29.3 ± 6.2	29.1 ± 6.2	0.03
Comorbidities, n (%)						
Hypertension	22,734 (71.2)	151,153 (57.4)	0.29	22,725 (71.2)	23,069 (72.3)	0.02
Hyperlipidemia	16,858 (52.8)	102,628 (39)	0.28	16,849 (52.8)	17,018 (53.4)	0.01
Neoplasms	13,945 (43.7)	89,574 (34.0)	0.20	13,937 (43.7)	14,178 (44.4)	0.02
Diabetes mellitus	10,346 (32.4)	60,828 (23.1)	0.21	10,337 (32.4)	10,244 (32.1)	0.01
Ischemic heart diseases	9,910 (31.1)	54,603 (20.7)	0.24	9,901 (31)	9,837 (30.8)	<0.001
Chronic lower respiratory diseases	10,405 (32.6)	51,275 (19.5)	0.30	10,396 (32.6)	10,400 (32.6)	<0.001
Overweight and obesity	9,087 (28.5)	47,833 (18.2)	0.25	9,082 (28.5)	8,977 (28.1)	0.01
Chronic kidney disease	6,899 (21.6)	38,844 (14.7)	0.18	6,891 (21.6)	6,661 (20.9)	0.02
Cerebrovascular diseases	5,074 (15.9)	27,950 (10.6)	0.16	5,068 (15.9)	4,859 (15.2)	0.02
Asthma	5,022 (15.7)	24,546 (9.3)	0.19	5,017 (15.7)	5,078 (15.9)	0.01
Chronic liver diseases	3,792 (11.9)	18,674 (7.1)	0.16	3,784 (11.9)	3,645 (11.4)	0.01
Pulmonary heart disease	3,364 (10.5)	12,098 (4.6)	0.23	3,355 (10.5)	3,065 (9.6)	0.03
Immunosuppressants	3,055 (9.6)	13,562 (5.2)	0.17	3,046 (9.5)	2,864 (9)	0.02
Nicotine dependence	2,319 (7.3)	14,426 (5.5)	0.07	2,317 (7.3)	2,152 (6.7)	0.02

Standardized difference (Std diff) < 0.1 is considered a small difference.

### 3.2 Primary outcomes following primary infection and reinfection

**Figure 2:**
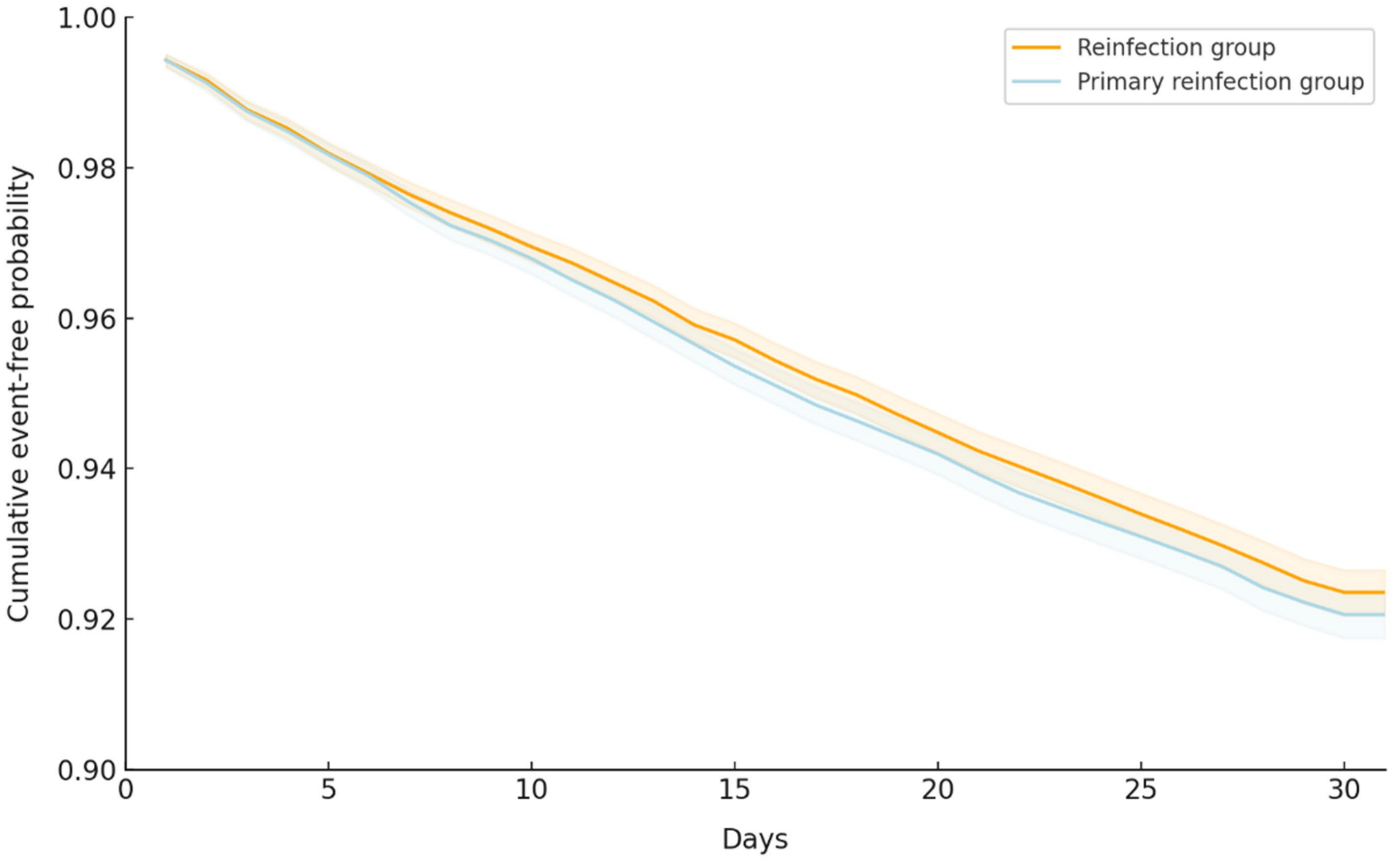
Kaplan–Meier time-to-event free curve for the primary composite outcome of patients with COVID-19 reinfection and primary infection.

Regarding the primary outcomes following the SARS-CoV-2 infections, the risk of composite outcomes, including ED visits, all-cause hospitalization, ICU admission, MV use, and mortality, was 7.15% (*n* = 2,281) in the reinfection group and 7.53% (*n* = 31,899) in the primary infection group. There was no significant difference between the groups (HR, 0.96; 95% CI, 0.91 to 1.02, *p* = 0.17; Table 2). Additionally, there was no significant difference in time-to-event-free survival between the reinfection and primary infection groups using Kaplan–Meier plots (log-rank *p* = 0.07; Figure 2).

**Table 2:** The hazard ratio for comparing matched between the reinfection and primary infection cohorts for the outcomes.

Outcomes	Patients with outcome		Hazard ratio (95%CI)	*p* value
	Reinfection group (*n* = 31,899)	Primary infection (*n* = 31,899)		
Primary outcome	2,281	2,403	0.96 (0.91 to 1.02)	0.17
Secondary outcomes				
Emergency department visits	1,353	1,334	1.03 (0.95 to 1.11)	0.49
All-cause hospitalization	1,071	1,157	0.94 (0.86 to 1.02)	0.14
Intensive care unit admission	69	76	0.92 (0.67 to 1.28)	0.62
Mechanical ventilation use	20	15	1.35 (0.69 to 2.64)	0.38
All-cause mortality	131	141	0.94 (0.74 to 1.20)	0.62

### 3.3 Subgroup analysis

**Figure 3:**
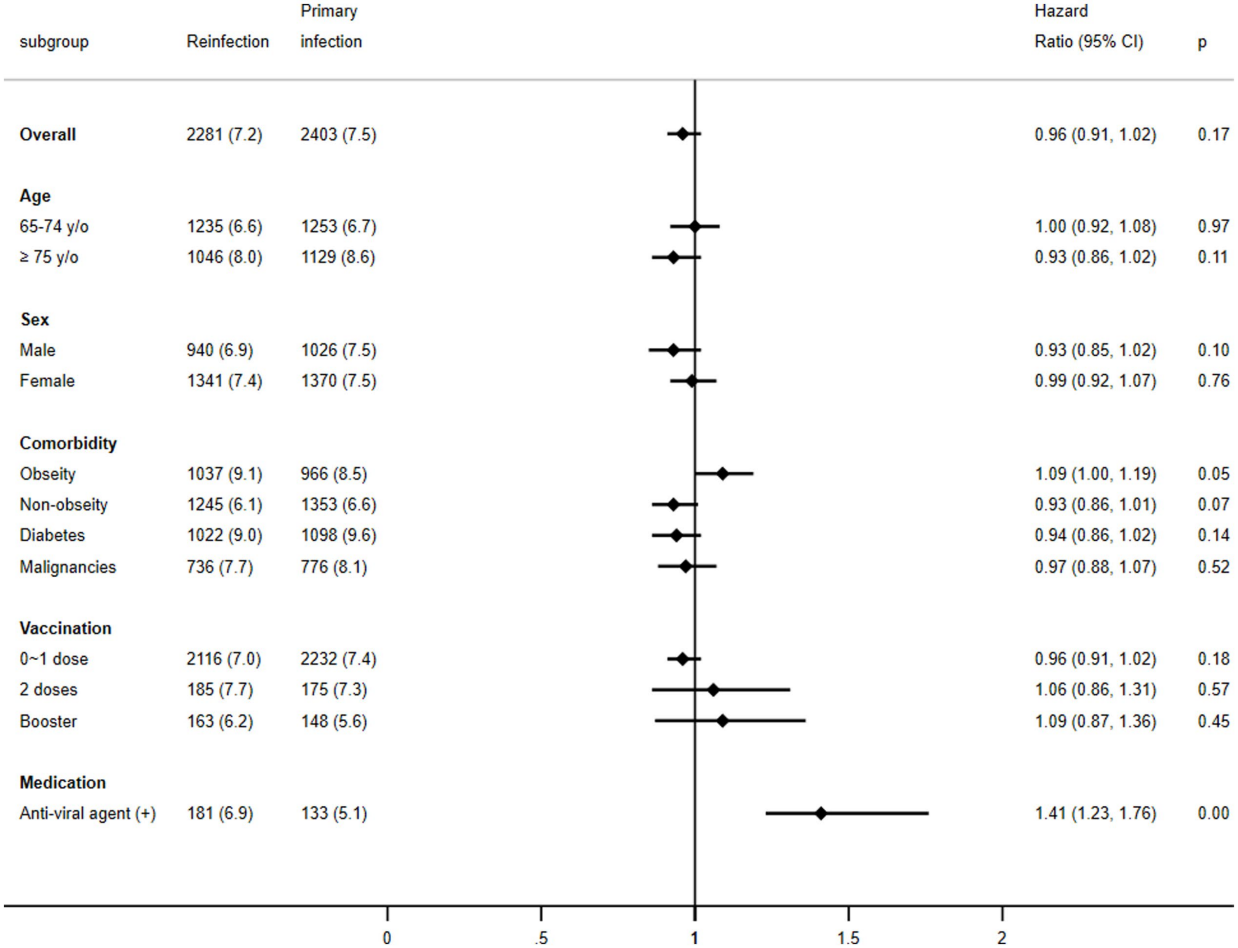
Forest plots of the primary outcome between COVID-19 reinfection and primary infection according to the subgroup.

Further subgroup analysis revealed a similar trend with no significant differences between the groups in the primary outcomes following the SARS-CoV-2 infections for the various subgroups. This included patients aged 65–74 years (HR, 1.00; 95% CI, 0.92 to 1.08, *p* = 0.97), those aged ≥75 years (HR, 0.93; 95% CI, 0.86 to 1.02, *p* = 0.11), males (HR, 0.93; 95% CI, 0.85 to 1.02, *p* = 0.10), females (HR, 0.99; 95% CI, 0.92 to 1.07, *p* = 0.76), individuals with obesity (HR, 1.09; 95% CI, 1.00 to 1.19, *p* = 0.05), those with diabetes mellitus (HR, 0.94; 95% CI, 0.86 to 1.02, *p* = 0.14), patients with malignancy (HR, 0.97; 95% CI, 0.88 to 1.07, *p* = 0.11), those receiving 0–1 vaccinations (HR, 0.96; 95% CI, 0.81 to 1.02, *p* = 0.18), individuals receiving 2 vaccinations (HR, 0.93; 95% CI, 0.86 to 1.31, *p* = 0.57), and those receiving booster vaccination (HR, 1.09; 95% CI, 0.87 to 1.36, *p* = 0.57). In contrast, among the subgroup of individuals receiving oral antiviral agents, the reinfection group was associated with a significantly higher risk than the primary infection group (HR, 1.41; 95% CI, 1.23 to 1.76, *p* < 0.001; Figure 3).

### 3.4 Secondary outcomes

Regarding secondary outcomes following the SARS-CoV-2 infections, there were no significant differences between the reinfection and primary infection group in terms of ED visit (HR, 1.03; 95% CI, 0.95 to 1.11, *p* = 0.49), all-cause hospitalization (HR, 0.94; 95% CI, 0.86 to 1.02, *p* = 0.14), ICU admission (HR, 0.92; 95% CI, 0.67 to 1.28, *p* = 0.62), MV use (HR,1.35 95% CI, 0.69 to 2.64 *p* = 0.38), all-cause mortality (HR, 0.94; 95% CI, 0.74 to 1.20, *p* = 0.62; Table 2).

## 4 Discussion

The alarming surge in COVID-19 cases, which is further exacerbated by reinfection, highlights the need to scrutinize the implications and outcomes of SARS-CoV-2 reinfection, especially among older adults. This study meticulously curated data from the TriNetX research network with the aim of unveiling and comparing the short-term outcomes between reinfection and primary infection among older adult patients who are characteristically at a heightened risk of severe COVID-19 progression.

Our finding from this large retrospective cohort study revealed no significant difference of composite outcome including ED visits, all-cause hospitalization, ICU admission, MV use, and mortality following the SARS-CoV-2 infections between the reinfection and primary infection among the older adult populations (HR, 0.96; 95% CI, 0.91 to 1.02, *p* = 0.17). In the subgroup analysis, outcomes were similar with respect to patient age, sex, commodity, and number of vaccinations. This could indicate that previous SARS-CoV-2 infections may not dramatically alter the risk of severe outcomes during a subsequent infection within this demographic, which diverges from the conclusions of preceding research outcomes.

Although the reinfection risk is relatively low among the older adult population, a higher risk of disease severity was found to be associated with older adults age and underlying comorbidities, in a previous study in the United Kingdom (15). Therefore, it is important to know the outcome of older adult patients with COVID-19 reinfection. Our study is the first to compare the outcomes between primary and reinfected older adult populations with similar baseline characteristics, after matching. This contributes a nuanced layer to our growing understanding of SARS-CoV-2 reinfection, particularly in the older adults.

Intriguingly, while our data did not indicate a universal increase in risk across all older adult patients with reinfection, the subgroup analysis revealed a peculiar exception for those administered antiviral agents. Previous studies (19, 20) revealed hybrid immunity offers stronger protection than immunity obtained from a previous infection or vaccination alone. Warth et al. revealed that vaccination of a previously infected patient induced a higher concentration of three types of antibodies, including anti-S, anti-N, and Angiotensin I Converting Enzyme 2 Receptor Binding Protein (ACE2-RBD)-blocking antibodies (20). However, the effectiveness against severe, critical, or fatal COVID-19 was inconclusive due to the small number of cases. In our study, the outcomes of the reinfected older adult population were not significantly different from those of the population that received at least two doses of vaccination. This implies that hybrid immunity may effectively prevent infection but does not affect disease severity. Most importantly, the previous antiviral treatment did not offer the benefits for the outcomes after reinfection. There are some speculations, including the use of antiviral agents, which may induce mutations in the virus genome during replication (21, 22), and the other possibility, including the patient who had antiviral treatment, mostly belonged to high risk, significant symptomatic, or severe disease during the primary infection. Whether these signals are a possible underlying comorbidity, variations in immune response, or perhaps allude to an aspect of the viral encounter that escalates the risk, remains a potent avenue for future investigations.

The strength of this study is that it included a large cohort based on the TriNetX platform, and the outcomes of interest were accurately recorded. However, this study has several limitations. First, coding or uncoding errors may have occurred, particularly in the presence of comorbidities. Second, although PSM was used to balance the baseline differences between these two groups of patients, the severity of the comorbidities used could not be evaluated using the claims database. In addition, geographical diversity and variability in healthcare systems and policies across the participating HCOs might have introduced heterogeneity, even though a robust PSM technique was employed to minimize confounding and selection biases. Finally, variable vaccination statuses and immunization schedules, in conjunction with the potential influence of various antiviral and therapeutic strategies, embed additional complexity in the interpretive framework.

## 5 Conclusion

This study provided updated information on the outcomes of older adult patients with COVID-19. Overall, the outcomes of reinfection did not differ from those of the primary infection, and this trend remained unchanged across most subgroups, including vaccination status, age, comorbidities, and sex. However, the use of antiviral agents for reinfection is not as effective as for primary infection.

## Data availability statement

The original contributions presented in the study are included in the article/supplementary materials, further inquiries can be directed to the corresponding authors.

## Ethics statement

The studies involving humans were approved by Chi Mei Medical Center, Tainan, Taiwan. The studies were conducted in accordance with the local legislation and institutional requirements. The ethics committee/institutional review board waived the requirement of written informed consent for participation from the participants or the participants' legal guardians/next of kin because retrospective study design based on TriNetX platform.

## Author contributions

S-FT: Conceptualization, Investigation, Writing – original draft. Y-WT: Data curation, Formal analysis, Investigation, Writing – original draft. J-YW: Data curation, Investigation, Methodology, Writing – original draft. T-HL: Data curation, Formal analysis, Investigation, Writing – original draft. M-HC: Writing – original draft. W-HH: Data curation, Formal analysis, Investigation, Validation, Writing – original draft. P-YH: Data curation, Formal analysis, Investigation, Writing – original draft. C-CL: Conceptualization, Writing – review & editing. C-KH: Conceptualization, Supervision, Writing – review & editing.
